# Supplementary material for: An In Vitro System for Evaluating Molecular Targeted Drugs Using Lung Patient-Derived Tumor Organoids
Source: Cells. 2019 May 20;8(5):481. doi: 10.3390/cells8050481 (PMC6562414; doi:10.3390/cells8050481)
Supplement: Supplementary file 1 [file cells-08-00481-s001.zip › Supplementary data/F-PDO_molecular targeted drugs_Supplementary Materials.docx]

Table S1. Anticancer agents used in the present study.

| Compound | Target | Supplier | Purity, % |
| --- | --- | --- | --- |
| GDC-0068 | AKT | MedChemExpress | 100 |
| Erlotinib | EGFR | Carbosynth | 100 |
| Osimertinib | EGFR | MedChemExpress | 100 |
| Rociletinib | EGFR | MedChemExpress | 100 |
| Lapatinib | EGFR，HER2 | LC Laboratories | 100 |
| Afatinib | EGFR，HER2 | Selleck Chemicals | 100 |
| ARRY-380 | HER2 | Selleck Chemicals | 96 |
| Varlitinib | HER2，EGFR | Selleck Chemicals | 98 |
| Binimetinib | MEK | Active Biochem | 100 |
| AZD 6244 | MEK | LC Laboratories | 100 |
| NVP-BKM120 | PI3K | AdooQ | 100 |
| Idelalisib | PI3K | AdooQ | 100 |
| GDC-0980 | PI3K，mTOR | MedChemExpress | 100 |
| Dabrafenib | Raf | AdooQ | 100 |
| Vemurafenib | Raf | Chemscene | 100 |
| Sorafenib | Raf，VEGFR，Ret，PDGFR | Selleck Chemicals | 92 |
| Nilotinib | ABL | Carbosynth | 100 |
| Ponatinib | ABL，Kit，Ret，FGFR | LC Laboratories | 100 |
| DCC-2036 | ABL，SRC，FLT3 | AdooQ | 100 |
| Ceritinib | ALK | Chemie Tek | 100 |
| Crizotinib | ALK，HGFR | LC Laboratories | 100 |
| Entrectinib | ALK，TrkA，TrkB，TrkC，ROS1 | MedChemExpress | 100 |
| Danusertib | Aurora | AdooQ | 93 |
| Obatoclax | Bcl | LC Laboratories | 100 |
| Ibrutinib | Btk | MedChemExpress | 100 |
| PAC-1 | Casepase | AdooQ | 100 |
| PHA-793887 | CDK | AdooQ | 100 |
| Dinaciclib | CDK | Cayman Chemical | 100 |
| GSK126 | EZH2 | AdooQ | 100 |
| Entinostat | HDAC | Carbosynth | 100 |
| Panobinostat | HDAC | Cayman Chemical | 98 |
| Belinostat | HDAC | Selleck Chemicals | 100 |
| PCI-34051 | HDAC | Selleck Chemicals | 98 |
| Tubastatin A | HDAC | Selleck Chemicals | 100 |
| Vismodegib | Hedgehog | LC Laboratories | 100 |
| Tivantinib | HGFR | MedChemExpress | 100 |
| Foretinib | HGFR，VEGFR，PDGFR，Kit，FLT3，Tie，Ron | Selleck Chemicals | 98 |
| Ganetespib | HSP90 | Selleck Chemicals | 100 |
| AGI-5198 | IDH1 | Calbio Chem | 100 |
| Vorasidenib | IDH1,IDH2 | Selleck Chemicals | 100 |
| AGI-6780 | IDH2 | MedChemExpress | 98 |
| OSI-906 | IGF | Chemie Tek | 100 |
| BMS-754807 | IGF | Chemscene | 99 |
| Ruxolitinib | JAK | Chemscene | 100 |
| Everolimus | mTOR | AdooQ | 98 |
| MLN-4924 | NAE | AdooQ | 100 |
| Olaparib | PARP | AdooQ | 100 |
| Tandutinib | PDGFR，Kit，FLT3 | LC Laboratories | 100 |
| Volasertib | PLK | Chemie Tek | 100 |
| Bortezomib | Proteasome | AdooQ | 100 |
| AZD 4547 | Raf | Active Biochem | 100 |
| RO-4929097 | Secretase | AdooQ | 100 |
| AZD 0530 | SRC，ABL | Chemscene | 100 |
| BX-795 | TBK，PDK，IKK | AdooQ | 97 |
| Nutlin-3 | Ubiquitin | KareBay Biochem | 100 |
| Brivanib | VEGFR，FGFR | AdooQ | 100 |
| Sunitinib | VEGFR，FGFR，PDGFR，Kit，FLT3 | Cayman Chemical | 97 |
| Regorafenib | VEGFR，Kit，Ret，FGFR，PDGFR | MedChemExpress | 100 |
| Lenalidomide | Thalidomide | AdooQ | 100 |
| Gemcitabine | DNA synthesis | Ark Pharm | 100 |
| Carfilzomib | Proteasome | Chemie Tek | 100 |
| Dexamethasone | Corticosteroid | Fujifilm Wako | 100 |
| Methotrexate | Dihydrofolate reductase | Fujifilm Wako | 100 |
| Etoposide | DNA topoisomerase | Fujifilm Wako | 100 |
| Melphalan | DNA alkylation | Fujifilm Wako | 100 |
| Temozolomide | DNA alkylation | Fujifilm Wako | 100 |
| Mitomycin C | DNA synthesis | Fujifilm Wako | 100 |
| Fluorouracil | DNA synthesis | Fujifilm Wako | 100 |
| Tacrolimus | Calcineurin | LC Laboratories | 100 |
| Rapamycin | mTOR | LC Laboratories | 100 |
| Elesclomol | Apoptosis | Selleck Chemicals | 100 |
| Alvespimycin | HSP90 | Selleck Chemicals | 100 |
| Vindesine | Tubulin | Sigma | 100 |
| Leflunomide | Dihydroorotate dehydrogenase | TCI | 100 |
| Decitabine | DNA demethylating | TCI | 100 |
| Carboplatin | DNA synthesis | TCI | 100 |
| Mycophenolic acid | Raf | TCI | 100 |
| Paclitaxel | Tubulin | TCI | 100 |

Table S2. Response of F-PDOs to anti-cancer agents (Molecular target drugs that inhibit the EGFR signal pathway.). IC_50_ and AUC values.

| Compound | RLUN5 | | RLUN16 | | RLUN21 | | RLUN21 + EGF | |
| --- | --- | --- | --- | --- | --- | --- | --- | --- |
|  | IC_50_ | AUC | IC_50_ | AUC | IC_50_ | AUC | IC_50_ | AUC |
| GDC-0068 | >20 | 1667 | 0.93 | 368 | 4.05 | 635 | 2.25 | 617 |
| Erlotinib | 8.96 | 1160 | >20 | 1905 | 0.20 | 723 | 1.48 | 408 |
| Osimertinib | 1.01 | 389 | 3.30 | 400 | 0.76 | 231 | 0.67 | 102 |
| Rociletinib | 4.96 | 655 | 8.05 | 934 | 1.57 | 472 | 2.23 | 363 |
| Afatinib | 0.53 | 176 | 1.10 | 141 | 0.04 | 468 | 0.08 | 108 |
| Lapatinib | >20 | 1681 | 11.66 | 1151 | 1.13 | 312 | 3.79 | 407 |
| ARRY-380 | >20 | 2061 | 19.38 | 1975 | 6.65 | 685 | 2.52 | 281 |
| Varlitinib | 11.30 | 1391 | 8.84 | 1280 | 0.78 | 853 | 1.68 | 487 |
| AZD 6244 | >20 | 1873 | >20 | 1323 | >20 | 1165 | 0.07 | 439 |
| Binimetinib | >20 | 2191 | >20 | 1303 | >20 | 1387 | 0.07 | 356 |
| Idelalisib | >20 | 2240 | >20 | 1970 | >20 | 2075 | >20 | 1950 |
| NVP-BKM120 | 3.39 | 412 | 1.73 | 226 | 9.77 | 1075 | 2.74 | 451 |
| GDC-0980 | 0.77 | 673 | 0.22 | 47 | 0.59 | 199 | 0.15 | 90 |
| Dabrafenib | >20 | 1958 | >20 | 1916 | 6.62 | 883 | 2.78 | 772 |
| Vemurafenib | >20 | 1704 | >20 | 1402 | 17.43 | 1374 | 2.16 | 246 |
| Sorafenib | 1.38 | 183 | 1.88 | 196 | 1.77 | 263 | 1.32 | 173 |

Table S3. Response of F-PDOs to anti-cancer agents (Other molecular target drugs.). IC_50_ and AUC values.

| Compound | RLUN5 | | RLUN16 | | RLUN21 | | RLUN21 + EGF | |
| --- | --- | --- | --- | --- | --- | --- | --- | --- |
|  | IC_50_ | AUC | IC_50_ | AUC | IC_50_ | AUC | IC_50_ | AUC |
| Dinaciclib | 0.01 | 3 | 0.01 | 1 | 0.01 | 7 | 0.01 | 1 |
| Bortezomib | 0.02 | 4 | 0.02 | 11 | 0.01 | 8 | 0.02 | 2 |
| Panobinostat | 0.03 | 7 | 0.01 | 0 | 0.02 | 113 | 0.01 | 26 |
| Volasertib | 0.01 | 12 | 0.01 | 63 | 0.03 | 407 | 0.01 | 165 |
| MLN-4924 | 0.12 | 43 | 0.49 | 352 | 0.15 | 306 | 0.04 | 46 |
| Tivantinib | 0.08 | 62 | 0.06 | 139 | 8.74 | 1017 | 0.07 | 274 |
| PHA-793887 | 0.22 | 86 | 0.37 | 215 | 0.95 | 252 | 0.11 | 109 |
| DCC-2036 | 0.91 | 98 | 2.16 | 231 | 0.69 | 101 | 0.97 | 116 |
| Belinostat | 1.02 | 122 | 0.59 | 63 | 0.24 | 342 | 0.13 | 41 |
| Ganetespib | 0.03 | 129 | 0.03 | 186 | 0.03 | 142 | 0.01 | 30 |
| Ponatinib | 1.98 | 198 | 0.69 | 85 | 0.43 | 60 | 0.10 | 27 |
| Regorafenib | 2.07 | 226 | 2.05 | 235 | 2.68 | 444 | 0.54 | 143 |
| Entinostat | 1.39 | 227 | 1.01 | 115 | 0.00 | 1588 | 1.22 | 188 |
| Olaparib | 1.29 | 241 | >20 | 1551 | >20 | 1205 | 4.87 | 899 |
| Danusertib | 0.94 | 259 | 0.28 | 354 | 0.29 | 589 | 0.09 | 233 |
| AGI-6780 | 3.44 | 420 | 3.77 | 449 | 10.00 | 1047 | 2.36 | 365 |
| Ceritinib | 4.68 | 476 | 1.69 | 197 | 2.13 | 295 | 1.00 | 100 |
| Entrectinib | 5.17 | 587 | 2.62 | 266 | 3.31 | 570 | 1.89 | 245 |
| Crizotinib | 4.89 | 596 | 1.26 | 157 | 2.49 | 297 | 0.42 | 123 |
| GSK126 | 10.60 | 1068 | 2.80 | 558 | >20 | 2036 | 12.54 | 1120 |
| Ibrutinib | 10.55 | 1070 | 7.55 | 869 | 1.34 | 927 | 0.55 | 242 |
| Everolimus | 7.94 | 1072 | 0.58 | 457 | >20 | 1520 | 0.01 | 474 |
| BX-795 | 10.95 | 1106 | 11.83 | 1170 | 9.33 | 1025 | 0.21 | 283 |
| Obatoclax | 9.70 | 1127 | 1.85 | 296 | 0.50 | 178 | 0.07 | 31 |
| PAC-1 | 11.48 | 1155 | 14.37 | 1432 | 16.05 | 1406 | 6.34 | 660 |
| Brivanib | 6.99 | 1172 | >20 | 1614 | 15.78 | 1656 | 2.67 | 476 |
| Tubastatin A | 14.81 | 1362 | 11.16 | 1180 | >20 | 1996 | 10.14 | 1070 |
| Foretinib | 15.95 | 1457 | 2.84 | 482 | 0.50 | 694 | 0.45 | 277 |
| Tandutinib | 15.88 | 1466 | 10.70 | 1112 | 18.92 | 1880 | 8.05 | 846 |
| Sunitinib | 15.06 | 1470 | 4.96 | 598 | 3.67 | 583 | 1.26 | 321 |
| OSI-906 | 17.45 | 1575 | 5.55 | 777 | >20 | 1343 | 7.18 | 903 |
| AZD 0530 | >20 | 1762 | 7.45 | 869 | 4.79 | 817 | 5.85 | 675 |
| BMS-754807 | 18.06 | 1792 | 0.12 | 288 | 5.01 | 987 | 0.10 | 451 |
| Nutlin-3 | >20 | 1915 | 19.03 | 1608 | >20 | 1824 | 19.31 | 2059 |
| AZD 4547 | 19.05 | 1985 | 9.03 | 987 | 19.72 | 1641 | 10.15 | 1008 |
| RO-4929097 | >20 | 1990 | >20 | 2120 | >20 | 1461 | >20 | 1672 |
| Ruxolitinib | >20 | 2014 | >20 | 1897 | >20 | 1124 | >20 | 1327 |
| PCI-34051 | >20 | 2046 | >20 | 1902 | >20 | 1821 | 18.56 | 1639 |
| AGI-5198 | >20 | 2063 | >20 | 2152 | >20 | 2392 | >20 | 2064 |
| Vismodegib | >20 | 2144 | >20 | 1826 | >20 | 1995 | 9.43 | 1104 |
| Vorasidenib | >20 | 2281 | >20 | 2081 | >20 | 1926 | >20 | 1244 |
| Nilotinib | >20 | 2546 | >20 | 2100 | >20 | 2277 | >20 | 1850 |

Table S4. Response of F-PDOs to anti-cancer agents (Chemotherapeutic drugs.). IC_50_ and AUC values.

| Compound | RLUN5 | | RLUN16 | | RLUN21 | | RLUN21 + EGF | |
| --- | --- | --- | --- | --- | --- | --- | --- | --- |
|  | IC_50_ | AUC | IC_50_ | AUC | IC_50_ | AUC | IC_50_ | AUC |
| Carfilzomib | 0.10 | 0 | 0.06 | 13 | 0.06 | 27 | 0.01 | 0 |
| Gemcitabine | 0.04 | 13 | >20 | 1043 | 0.02 | 457 | 0.00 | 109 |
| Paclitaxel | 0.00 | 16 | 0.00 | 81 | >20 | 1877 | 0.00 | 288 |
| Mitomycin C | 0.03 | 34 | 0.13 | 176 | 1.94 | 360 | 1.30 | 201 |
| Vindesine | 0.00 | 39 | 0.00 | 193 | >20 | 1165 | 0.00 | 310 |
| Methotrexate | 0.03 | 44 | 0.19 | 449 | 7.54 | 1027 | 0.15 | 377 |
| Etoposide | 0.20 | 62 | 0.41 | 309 | 2.19 | 611 | 0.32 | 377 |
| Melphalan | 0.85 | 141 | 3.47 | 715 | >20 | 1844 | 6.55 | 899 |
| Alvespimycin | 0.12 | 150 | 0.03 | 157 | 0.67 | 240 | 0.06 | 83 |
| Carboplatin | 2.13 | 326 | 15.65 | 1476 | 7.13 | 944 | 5.12 | 693 |
| Decitabine | 1.32 | 354 | 5.31 | 951 | >20 | 1067 | 0.07 | 608 |
| Fluorouracil | 1.73 | 373 | 3.91 | 801 | 5.77 | 932 | 0.77 | 466 |
| Mycophenolic acid | 2.36 | 785 | 1.97 | 634 | >20 | 1385 | 0.56 | 385 |
| Elesclomol | >20 | 1602 | >20 | 2066 | >20 | 1246 | >20 | 1590 |
| Leflunomide | >20 | 1923 | >20 | 1745 | >20 | 1927 | 7.95 | 1051 |
| Dexamethasone | >20 | 1988 | >20 | 2015 | 0.01 | 828 | 0.01 | 849 |
| Lenalidomide | >20 | 2001 | >20 | 2028 | >20 | 1897 | >20 | 1662 |
| Rapamycin | 19.09 | 2002 | 0.05 | 433 | >20 | 1011 | 0.00 | 339 |
| Tacrolimus | >20 | 2011 | >20 | 2240 | >20 | 1525 | 14.87 | 1184 |
| Temozolomide | >20 | 2121 | >20 | 2254 | >20 | 2033 | >20 | 1882 |

Video S1: video of a cell cluster of RLUN21. Ki67-expression images prepared using an anti-Ki67 antibody (green). DNA stained with DAPI (blue). Magnification: ×30.
